# Selective Detection of Carbohydrates and Their Peptide Conjugates by ESI-MS Using Synthetic Quaternary Ammonium Salt Derivatives of Phenylboronic Acids

**DOI:** 10.1007/s13361-014-0857-4

**Published:** 2014-04-01

**Authors:** Monika Kijewska, Adam Kuc, Alicja Kluczyk, Mateusz Waliczek, Aleksandra Man-Kupisinska, Jolanta Lukasiewicz, Piotr Stefanowicz, Zbigniew Szewczuk

**Affiliations:** 1 Faculty of Chemistry, University of Wroclaw, Wroclaw, Poland; 2 Department of Immunochemistry, Ludwik Hirszfeld Institute of Immunology and Experimental Therapy, Polish Academy of Sciences, Wroclaw, Poland

**Keywords:** Quaternary ammonium salt, Mass spectrometry, Solid phase synthesis, Amadori products, Non-enzymatic glycation, Sugars, Phenylboronic acids

## Abstract

We present new tags based on the derivatives of phenylboronic acid and apply them for the selective detection of sugars and peptide-sugar conjugates in mass spectrometry. We investigated the binding of phenylboronic acid and its quaternary ammonium salt (QAS) derivatives to carbohydrates and peptide-derived Amadori products by HR-MS and MS/MS experiments. The formation of complexes between sugar or sugar-peptide conjugates and synthetic tags was confirmed on the basis of the unique isotopic distribution resulting from the presence of boron atom. Moreover, incorporation of a quaternary ammonium salt dramatically improved the efficiency of ionization in mass spectrometry. It was found that the formation of a complex with phenylboronic acid stabilizes the sugar moiety in glycated peptides, resulting in simplification of the fragmentation pattern of peptide-derived Amadori products. The obtained results suggest that derivatization of phenylboronic acid as QAS is a promising method for sensitive ESI-MS detection of carbohydrates and their conjugates formed by non-enzymatic glycation or glycosylation.

**Electronic supplementary material:**

The online version of this article (doi:10.1007/s13361-014-0857-4) contains supplementary material, which is available to authorized users.

**Figure:**
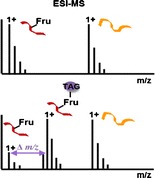
ᅟ

## Introduction

Glycation, one of the post-translational modifications of proteins, is a nonenzymatic reaction between sugar aldehyde or ketone and the amino groups of proteins. In the early stage of glycation, the intermediates leading to the Amadori compounds are formed [1]. Glucose-induced damage is not limited to diabetic patients; glycation also affects physiological aging and neurodegenerative diseases such as Alzheimer's disease and amyotrophic lateral sclerosis [2]. The modifications are formed at very low concentrations, which limit detection sensitivity of these modifications by conventional mass spectrometry methods. The MS analysis is also affected by the insufficient ionization efficiency of these peptides. The problem of selective detection of post-translational modifications, including glycation, was widely discussed in literature [3–9]. The direct sequencing of the peptide-derived Amadori products by collision induced dissociation (CID) is non-efficient because the CID spectrum of a glycated peptide reveals the characteristic pattern of neutral losses (several molecules of water, formaldehyde, and finally the whole hexose moiety) [3–5]. The abundance of peptide backbone fragments is relatively low and the sequence coverage is not sufficient to reconstruct the sequence of the studied peptide. Therefore, new techniques of ECD and ETD have been applied for the sequencing of the Amadori products [6, 7]. Another approach to the analysis of the primary structure of these compounds was based on a combination of enzymatic hydrolysis with mass spectrometry [8, 9]. Because of a very low concentration of modified proteins and peptides, new methods of selective detection based on isotopic labeling (^13^C or ^18^O) combined with mass spectrometry were researched [10, 11]. Another approach to overcome the low concentration of analyzed compounds is the application of sample preconcentration, frequently based on affinity methods. In case of LC-MS analysis of glycated peptides, borate affinity columns based on the specific interaction between phenylboronic acid derivatives and diols in sugar moieties have been used [12, 13].

It has been known for nearly 50 years that borate ion will complex with diols in solution forming borate esters [14]. The polyol structures of carbohydrates in glycopeptides can reversibly form anionic borate esters in the presence of tetrahydroxyborate ion, B(OH_4_)^–^ at alkaline pH (pH 8–10), which might be effectively used to distinguish glycopeptides from peptides [15]. The use of this reaction in combination with CE and HPLC has been reported for the separation of individual oligosaccharides released from glycoproteins and peptides with glycan variations, carbohydrates, and Amadori products [16–18]. Moreover, Chen and coworkers have demonstrated that surface plasmon resonance (SPR) can successfully detect and quantify the binding of HbA1c to phenylboronic acid monolayer by the specific interaction. This methodology may prove to be useful in clinical diagnosis and serve as an assay for HbA1c binding ability [19].

The reversible covalent association of phenylboronic acids and 1,2-*cis*-diols, catechols, or 2-hydroxycarboxylates to form cyclic boronic esters is one of the most explored interactions in molecular recognition [20]. In combination with salt bridges and hydrogen bonding, markers based on boronic acids have been developed for citrate [21], glucose-6-phosphate [22], and heparin [23]. Recently, a synthetic library of simple bis-boronic acid-based receptors with various spacers was used for the sensing of ginsenosides. The incorporation of two boronic acids allowed the pairing of two indicators, which can simultaneously bind the receptors or two saccharides within the ginsenosides. Moreover, the assay reported should be applicable to the analysis of other large saccharide-based natural products [24].

Although there is much written in the literature on the investigation of structures of borate polyol complexes by nuclear magnetic resonance spectroscopy (NMR) [25–28] and potentiometry [28], particularly with carbohydrates, there have been only a few reports on the use of mass spectrometry to investigate borate complexes. Two different techniques for the ionization of sample, fast atom bombardment (FAB) and negative ion electrospray (ESI), were investigated to confirm a number of boronic acid molecules, both showing excellent results [29]. Negative ion-mode FAB-MS was also used for the analysis of the structure of methyl β-D-apiofuranoside–borate complex [30]. Ackloo et al. have applied the concept of boric acid complexation to explore structural analysis of isomeric polyols using negative ion ESI-MS in conjunction with CID of the complexes [31]. Negative-ion-mode ESI-MS/MS is widely used for the analysis of the structures of borate complexes with different carbohydrates [32, 33]. In our recent work, we proposed an alternative method for selective detection of glycated peptides by mass spectrometry based on the borate complexes [34]. We presented the experiments on the formation and CID of borate complexes of peptide-derived Amadori products. MS experiments were performed in positive ion mode, which greatly facilitated the analysis of the MS/MS spectra. Our results indicate that the complexation of the borate anion by a glycated peptide may be used to distinguish glycated and non-glycated peptides; moreover, the complex stabilizes the hexose moiety attached to the lysine side chain, which reduces the neutral losses and simplifies CID spectrum [34].

In this work, we expanded our research on interactions of peptide-derived Amadori products and carbohydrates with phenylboronic acid and phenylboronic acid derivatives. We designed and synthesized new tags based on the combination of phenylboronic acid and quaternary ammonium salt and used them for selective detection of sugars and sugar-peptide conjugates, taking into account that incorporation of QAS into peptide chain significantly improves the efficiency of ionization of these compounds by mass spectrometry [35]. We performed the ESI-MS and ESI-MS/MS experiments using the model glycated peptides and biological samples to confirm the applicability of the selective detection method presented in this work. Our results indicate that complexes of glycated peptides and phenylboronic acid or phenylboronic acid derivatives also stabilize the sugar moiety in CID experiments. It could be expected that application of the new tags may improve selective and sensitive detection of carbohydrates or polysaccharides in positive ion mode in mass spectrometry.

## Experimental

### Reagents

All solvents and reagents were used as supplied. Fmoc amino acid derivatives were purchased from NovaBiochem (as Merck, Darmstadt, Germany). O-(6-chlorobenzotriazol-1-yl)-*N,N,N′,N′*-tetramethyluronium tetrafluoroborate (TCTU), the MBHA-Rink amide resin (0.67 mmol/g), and trifluoroacetic acid (TFA) were obtained from IrisBiotech (Marktredwitz, Germany). DABCO (1,4-diazabicyclo[2.2.2]octane), 4-(dimethylamino)phenylboronic acid, and 4-carboxyphenylboronic acid for the synthesis of quaternary ammonium salts (QAS) and solvents for peptide synthesis: *N,N*-dimethylformamide (DMF), dichloromethane (DCM), and *N*-ethyldiisopropylamine (DIEA) were obtained from Sigma Aldrich (St. Louis, MO, USA); iodoacetic acid from Merck (Darmstadt, Germany); *N,N*′-diisopropylcarbodiimide (DIC) and triisopropylsilane (TIS) from Fluka (as St. Louis, MO, USA). HPLC grade solvents used for purification [acetonitrile (MeCN) and methanol (MeOH)] were from Sigma Aldrich. Carbohydrates were obtained from Prolabo (Gdańsk, Poland) (D-arabinose), Fluka (D-ribose), ChemPur (D-glucose), Windsor Laboratory (D-glucuronic acid), Fluka (D-2-deoxyribose), Sigma-Aldrich (*N*-acetylglucosamine), Fluka (saccharose). Ammonium carbonate was purchased from Sigma Aldrich, the pH of the 50 mM buffer was adjusted to 9.0 with ammonia solution.

The oligosaccharide of lipopolysaccharide containing one RU linked to Hep-Kdo disaccharide was isolated from *Hafnia alvei* (Polish Collection of Microorganisms) PCM 1200 lipopolysaccharide (LPS) in the Department of Immunochemistry, Ludwik Hirszfeld Institute Immunology and Experimental Therapy, Polish Academy of Sciences [36].

### Glycated Peptides

The mono- and diglycated fragments of BSA [H-Asp-Thr-Glu-Lys-Gln-Ile-Lys(Fru)-Lys-Gln-Thr-OH, H-Asp-Thr-Glu-Lys(Fru)-Gln-Ile-Lys(Fru)-Lys-Gln-Thr-OH] and the model peptide H-Ala-Lys(Fru)-Ala-Phe-OH were prepared on solid support using the novel lysine derivative (N^α^-9-fluorenylmethoxycarbonyl-N^ε^-*tert*-butyloxycarbonyl-N^ε^-(2,3:4,5-di-*O-*isopropyliden-1-deoxy-β-D-fructopyranose-1-ylo)lysine, Fmoc-Lys(*i,i-*Fru,Boc)-OH. The details of the synthetic procedure were given in our previous paper [8].

Fmoc-Lys(Fru)-OH (N^α^-9-fluorenylmethoxycarbonyl-N^ε^-(1-deoxy-β-D-fructopyranose-1-ylo)lysine) was obtained by deprotection of Fmoc-Lys(*i,i-*Fru,BOC)-OH with TFA containing 5% of water. After 8 h, TFA was evaporated and the reaction product was used for MS experiments without further purification.

### Preparation of the Hydrolysate of Glycated Ubiquitin

The glycation of ubiquitin followed by the enzymatic hydrolysis of the glycation product were performed according the procedures described previously [6].

### Synthesis of Derivatives of Phenylboronic Acid and QAS

Products were prepared by manual solid-phase synthesis techniques, using the standard Fmoc synthetic procedure. The methodology of derivatization of lysine residues by DABCO in peptides on solid support was similar to the one presented by us previously [37].

**Figure 1:**
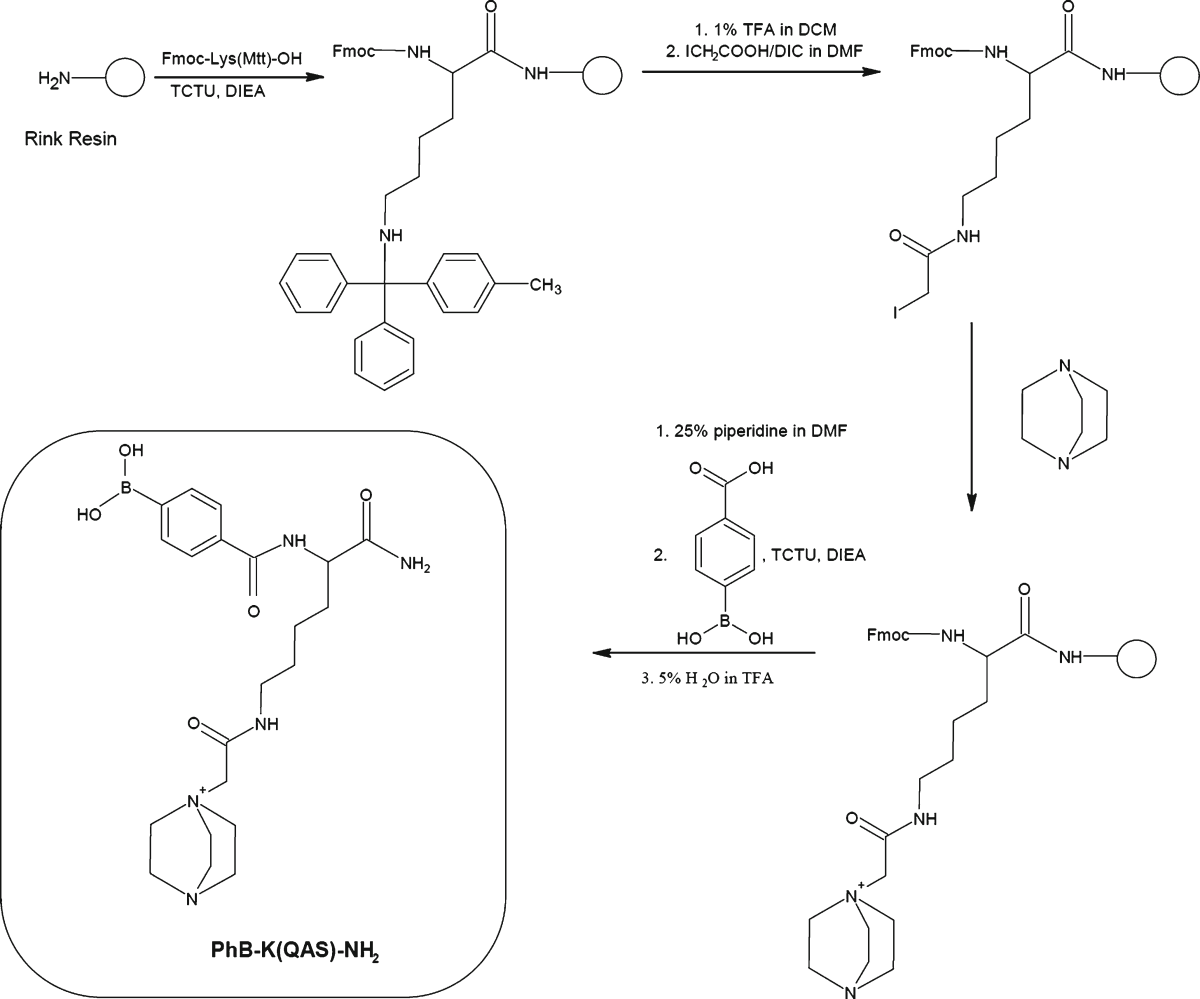
Synthesis of PhB-K(QAS)-NH_2_

Synthesis of PhB-K(QAS)-NH_2_ (TAG1): After attaching Fmoc-Lys(Mtt)-OH to the Rink resin, the Mtt protecting group was removed by using 1% solution of TFA in DCM (3 × 2 min, 3 × 10 min, 3 × 2 min) and the *ε*-amino group of the lysine residue was derivatized on solid support to form QAS. Then the peptidyl resin (30 mg, 0.02 mmol) was washed with DCM (3 × 1 min), DCM/DMF (1:1; vol:vol, 1 min), 5% DIEA in DMF (3 × 1 min), and DMF (7 × 1 min). The mixture of iodoacetic acid (18.7 mg, 0.1 mmol) and DIC (15.6 μL 0.1 mmol) dissolved in DMF (0.5 mL) was added and the reaction was allowed to proceed for 90 min with exchange of reagents solution after each 30 min. Then DABCO (45.1 mg, 0.4 mmol), dissolved in DMF (0.5 mL) was added to the reaction vessel and mixed for 12 h. After completing the synthesis of the QAS, the N-terminal Fmoc group was removed using the 25% solution of piperidine in DMF, and 4-carboxyphenylboronic acid (10 mg, 0.06 mmol) was attached, using TCTU (21 mg, 0.06 mmol) and DIEA (21 μL, 0.12 mmol) for 2 h at room temperature. The product was cleaved from the resin with the mixture of TFA/TIS/H_2_O (95:2.5:2.5; vol:vol:vol) for 2 h at room temperature and precipitated with cold diethyl ether (see Figure 1).

Yield: 52%, HPLC: Rt (min): 11.9 (conditions for HPLC are given in the Experimental section, *Purification*).

HR-MS (ESI-FT): *m/z*: [M]^+^ Calcd. for C_21_H_33_N_5_O_5_B 446.256; Found: 446.257.

**Figure 2:**
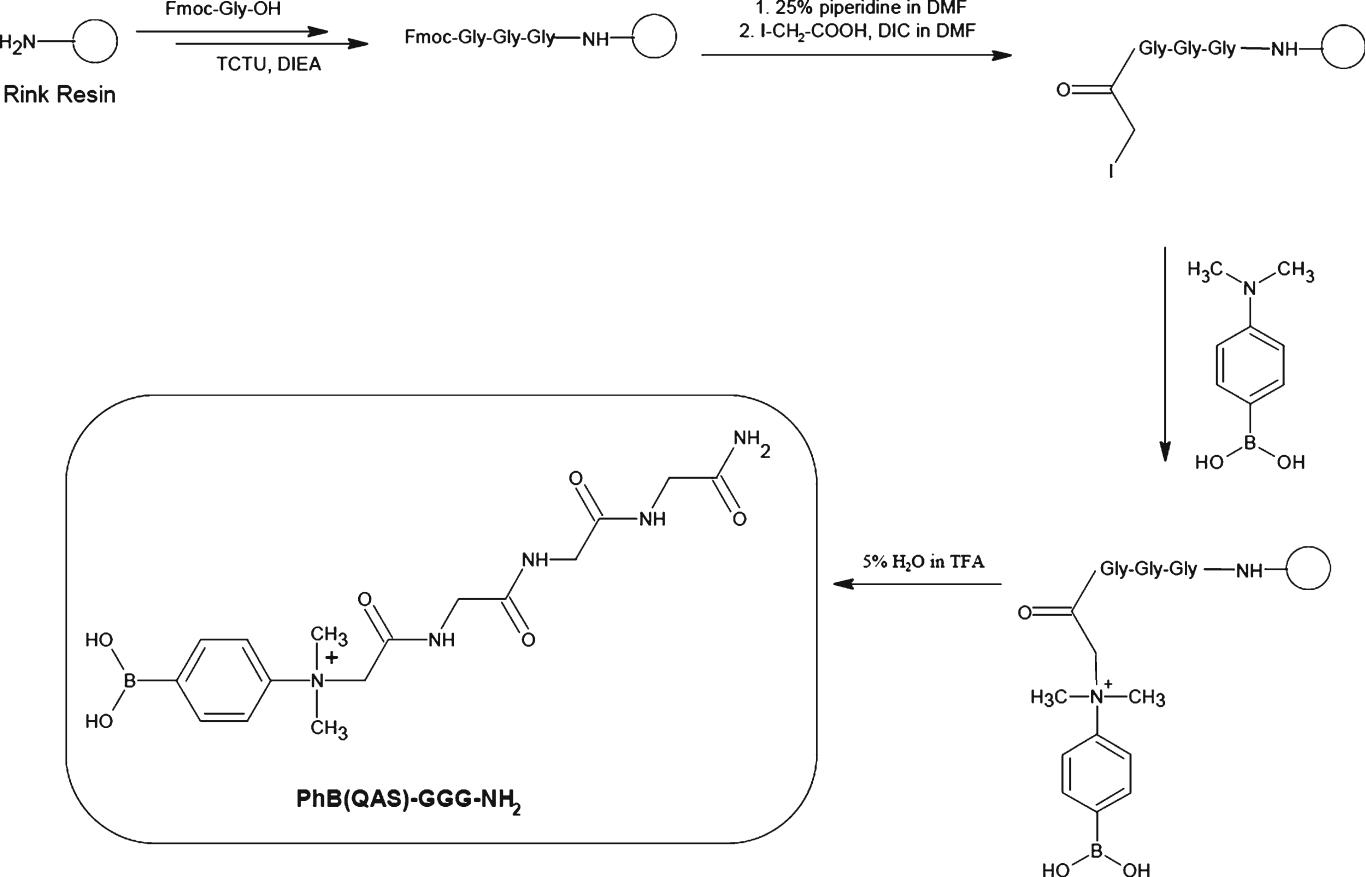
Synthesis of PhB(QAS)-GGG-NH_2_

Synthesis of PhB(QAS)-GGG-NH2 (TAG2): Peptides were prepared on solid support, using the Rink resin and standard Fmoc synthetic procedure. After synthesis of Fmoc-Gly-Gly-Gly-Rink, the *α*-amino group of glycine residue was derivatized on solid support using 4-(dimethylamino)phenylboronic acid to form QAS. The Fmoc protecting group was removed by using 25% solution of piperidine in DMF (1 × 3 min, 1 × 17 min). After the peptidyl resin (30 mg, 0.02 mmol) was washed with DMF (7 × 1 min), the mixture of iodoacetic acid (18.7 mg, 0.1 mmol) and DIC (15.6 μL, 0.1 mmol), dissolved in DMF (0.5 mL), was added and the reaction was allowed to proceed for 90 min with exchange of reagents solution after each 30 min. Then 4-(dimethylamino)phenylboronic acid (66.3 mg, 0.4 mmol), dissolved in DMF (0.5 mL), was added to the reaction vessel and mixed for 24 h. After incubation, the peptidyl resin was washed with DMF (7 × 1 min) and the product (PhB(QAS)-GGG-NH_2_) was cleaved from the resin using TFA/H_2_O/TIS (95:2.5:2.5, vol/vol) for 2 h at room temperature and precipitated with cold diethyl ether (see Figure 2).

Yield: 96%, HPLC: Rt (min): 7.1 (conditions for HPLC are given in the Experimental section, *Purification*).

HR-MS: (ESI-FT): *m/z*: [M]^+^ Calcd. for C_16_H_25_N_5_O_6_B 394.190; Found: 394.189.

### Purification

The crude products (QAS-phenylboronic acid derivatives) were analyzed using a Thermo Separation HPLC system with a UV detection (210 nm) on a Vydac Protein RP C18 column (4.6 × 250 mm, 5 μm), with a gradient elution of 0%–80% B in A (A = 0.1% TFA in water; B = 0.1% TFA in acetonitrile/H_2_O, 4:1) for 40 min (flow rate 1 mL/min). The main reaction product was purified by a preparative reversed-phase HPLC on a Tosoh (Tosoh Tokyo, Japan) TSKgel with an ODS-120 T column (21.5 mm × 300 mm), using a linear gradient 10%–20% of B for 40 min (PhB-K(QAS)-NH_2_) and 2%–12% of B for 40 min (PhB(QAS)-GGG-NH_2_), flow rate 7.0 mL/min, UV detection at 220 nm. The fractions were collected and lyophilized. The identities of the products were confirmed by MS analysis using Bruker Apex-Qe (Bremen, Germany) Ultra 7 T FT-ICR mass spectrometer equipped with an electrospray ionization source.

### Complex Formation

Samples of carbohydrate mixtures, Fmoc-Lys(*i*-Fru)-OH, glycated peptides, glycated ubiquitin hydrolysate, or core oligosaccharide were prepared in water with final concentration of 1 mg/mL. The aliquots (10–20 μL) were diluted by 50 mM ammonium carbonate buffer (500 μL). Then, 5 μL of phenylboronic acid solution (PhB, 1 mg in 1 mL of MeOH) or phenylboronic acid derivatives – 2 μL PhB-K(QAS)-NH_2_ (TAG1)(0.6 mg in 1 mL of H_2_O) or 3 μL PhB(QAS)-GGG-NH_2_ (TAG2)(0.6 mg in 1 mL of H_2_O) was added. After 1 min incubation, the sample was diluted with methanol (1:1, vol:vol) and subjected to ESI-MS experiment. All samples were also analyzed in ammonium borate buffer methanol mixture without complexing reagents.

### Mass Spectrometric Measurements

The samples were analyzed using micrOTOF-Q and Apex-Qe Ultra 7 T mass spectrometers (Bruker Daltonics). The micrOTOF-Q instrument equipped with an ESI source with ion funnel, was operated in positive or negative ion mode and calibrated before each analysis with the Tunemix mixture (Bruker Daltonics) in a quadratic method. In MS/MS experiments, the collision energy (5–40 eV) was optimized for the best fragmentation. Argon was used as a collision gas. The spectra were recorded in ammonium carbonate buffer (pH 9.0) at the peptide concentration of 0.5 μM.

The FT-ICR (Fourier transform ion cyclotron resonance) Apex-Qe Ultra 7 T instrument, equipped with an ESI source, was operated in the positive or negative ion mode. Analyte solutions were introduced at a flow rate of 3 μL/min. The instrument was calibrated before each analysis with the Tunemix mixture (Bruker Daltonics) in a quadratic method. The instrument parameters were as follows: scan range: 100–1600 *m/z*; drying gas: nitrogen; temperature: 200ºC; potential between the spray needle and the orifice: 4.5 kV. In the MS/MS experiments, precursor ions were selected on the quadrupole and subsequently fragmented in the hexapole collision cell. Argon was used as a collision gas. Obtained fragments were directed to the ICR mass analyzer and registered as an MS/MS spectrum. The collision voltage was optimized for the best fragmentation pattern.

For MS spectra analysis, a Bruker Compass DataAnalysis 4.0 software was used. A sophisticated numerical annotation procedure (SNAP) algorithm was used for finding peaks. All obtained signals had mass accuracy error in the range of 5 ppm. Spectra (positive ion mode) were recorded using a 50:50 acetonitrile–water mixture containing 0.1% HCOOH for standard analysis (e.g., confirmation of structure of obtained peptides and tags).

## Results and Discussion

### Complex Formation Using Phenylboronic Acid

The series of model synthetic derivatives (glycated peptides and Fmoc-Lys(Fru)-OH) as well as peptic fragments of ubiquitin were tested for their affinity towards the phenylboronic acid. In our previous work, we performed similar experiments to examine the stability of complexes of Amadori products with borate ion in mass spectrometry. We showed that the complexes are stable in the basic (pH >9) water-methanol solution [34]. Moreover, the attachment of borate ion to the fructosamine moiety stabilizes the sugar-bearing side chain in glycated peptides, which simplifies the analysis of CID spectra. The distance between the peak corresponding to the glycated peptide and its borate complex is only 8 Da, which may complicate interpretation of multiply charged ions because of the overlap of signals.

**Figure 3:**
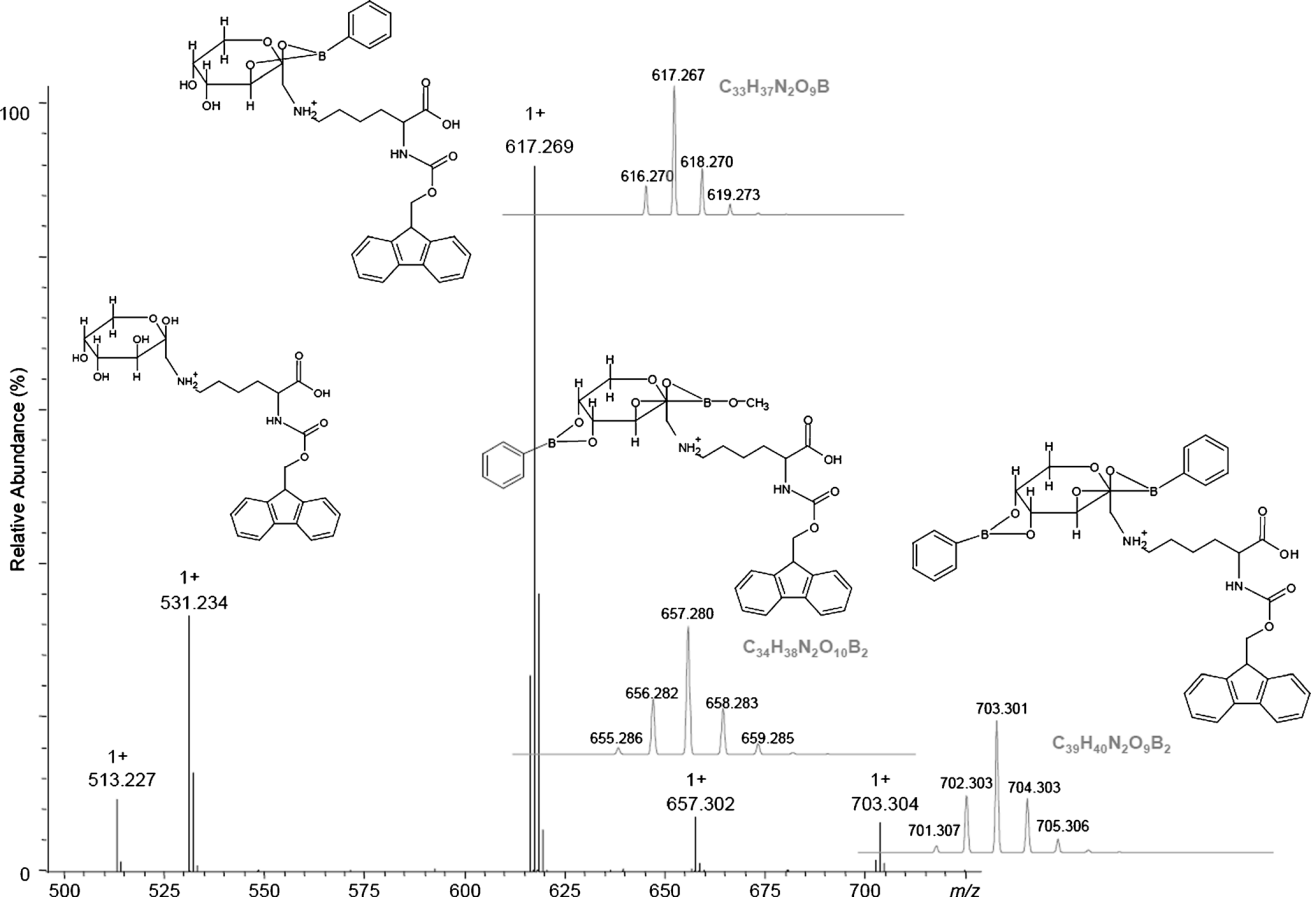
ESI-MS spectrum of the Fmoc-Lys(Fru)-OH complexed with phenylboronic acid (positive ion mode) (simulated isotopic patterns for proposed molecular formula of investigated compounds are shown)

To facilitate the interpretation of mass spectra, we used phenylboronic acid for the complex formation. The mixture of Fmoc-Lys(Fru)-OH and phenylboronic acid was incubated in ammonium carbonate buffer at the pH 9.0 (the details are included in Experimental). After 1 min the sample was diluted with methanol and analyzed using ESI-MS. The MS spectrum presented in Figure 3 shows a series of complexes containing phenylboronate anion. The most abundant complex contains one phenylboronate anion and one molecule of Fmoc-Lys(Fru)-OH, however, a complex containing two phenylboronate ions was also observed. The stoichiometry of these complexes is proven by their isotopic patterns. The parent ions at *m/z* 531.234, 617.269, and 703.304 were subjected to CID experiments (Supplemental Figures S1, S2, S3). The CID experiments were performed at the same range of collision energy of 5–35 eV to check the stability of the formed complex. In the MS spectrum of free glycated lysine, the characteristic neutral losses are observed (Supplemental Figure S1). The fragmentation of the peak corresponding to the complex of Fmoc-Lys(Fru)-OH with phenylboronic acid is characterized by a low abundance of ions formed by the elimination of water, but the ion formed by formaldehyde elimination is observed, although only after elimination of phenylboronate ion. In this case, the elimination of phenylboronate ion could be observed in mass spectrometry experiments (ion 447.193 *m/z* – Supplemental Figure S2). In our previous research, the collision energy required to obtain characteristic neutral losses for complex Fmoc-Lys(Fru)-OH with borate ion was lower [34].

The model glycated peptide H-Ala-Lys(Fru)-Ala-Phe-OH was also incubated with phenylboronic acid. The representative MS spectrum for the complex of this glycated peptide and phenylboronic acid is shown in Supplemental Figure S4. The peak at *m/z* 598.31 corresponds to the protonated peptide H-Ala-Lys(Fru)-Ala-Phe-OH, whereas the proposed structure for the ion at *m/z* 684.35 is presented in the Supplemental Figure S4. The *m/z* value and the characteristic isotopic pattern for this signal are in good agreement with the molecular formula C_33_H_46_N_5_O_10_B^+^. The parent ion at *m/z* 684.35 was subjected to MS/MS fragmentation (Supplemental Figure S5). The fragmentation of this ion was compared with results obtained for the protonated free glycated peptide (published previously [34]). The CID experiments were performed at a range of the collision energy of 5–45 eV to check the stability of the formed complex. The fragmentation spectrum of the complex of the model glycated peptide with phenylboronate is characterized by low abundance of ions formed by the elimination of water, and a low abundance peak corresponding to the ion formed by the elimination of a whole hexose moiety is observed (*m/z* 436.265). However, the fragmentation spectrum is dominated by the series of b and y ions covering the whole sequence of the peptide. In our previous work, focused on the study of stability of modified peptide–borate complex, the elimination of formaldehyde and a whole hexose moiety was not observed. We attributed the stability of the obtained complex to the stabilization of aminofructose moiety, which may be related to the crosslinking of the hexose with formation of a multi-ring structure [34] and the subsequent decrease in basicity (and, consequently, the protonation level) of the nitrogen attached to hexose moiety [38]. The analysis of the spectrum of the H-Ala-Lys(Fru)-Ala-Phe-OH peptide complexed with phenylboronic acid revealed that only two hydroxyl groups are involved in phenylboronic complexes, which may explain the lower collision energy required to fragment and eliminate the sugar moiety. Although the stabilization of hexose moiety is lower, it is still sufficient to indicate the modification site.

**Figure 4:**
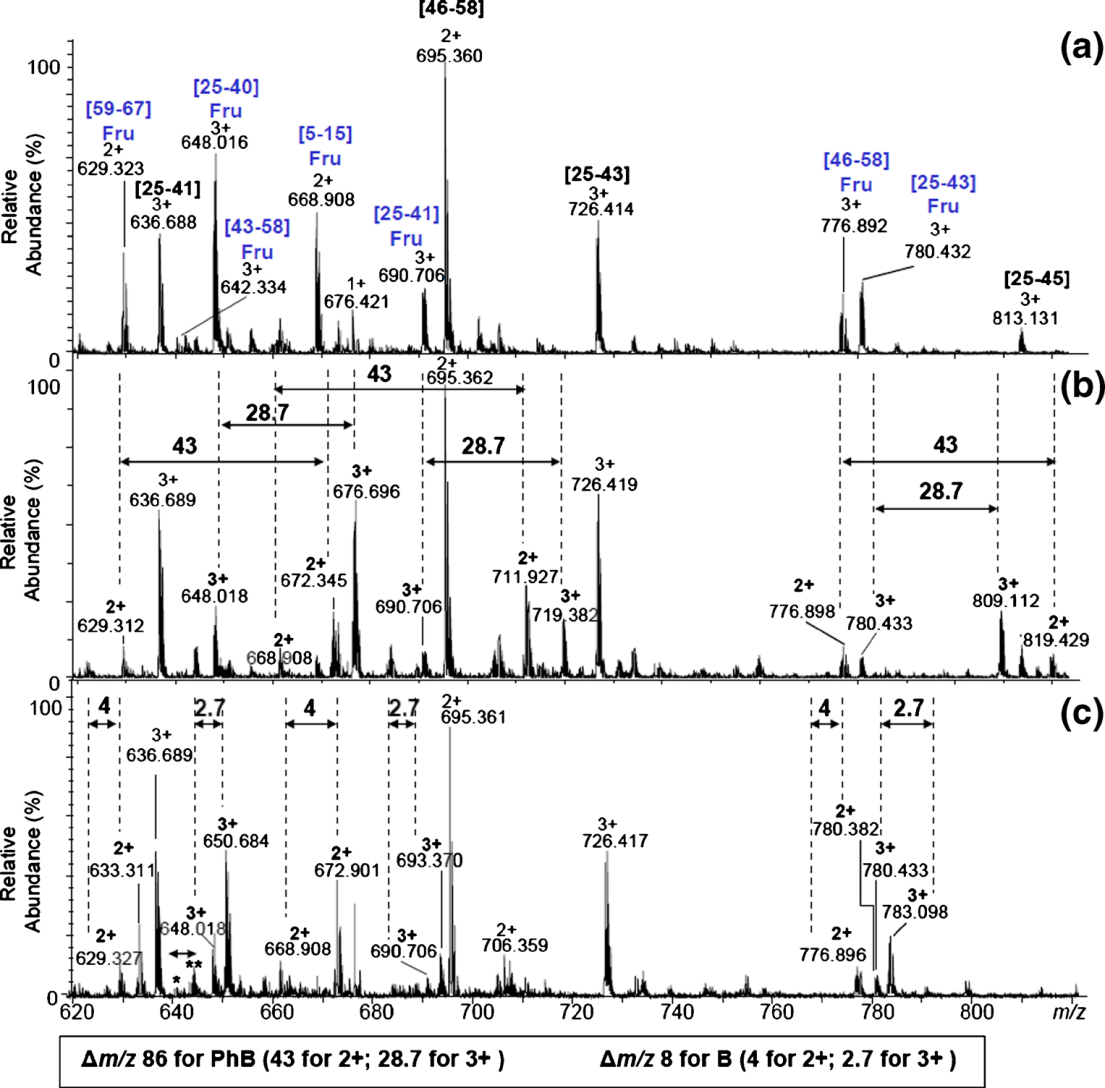
(**a**) ESI-MS spectrum of the glycated ubiquitin hydrolysate (positive ion mode); (**b**) ESI-MS spectrum of the glycated ubiquitin hydrolysate complexed with phenylboronic acid (PhB) (positive ion mode); (**c**) ESI-MS spectrum of the glycated ubiquitin hydrolysate complexed with bogate (B) (positive ion mode); *glycated peptide [43-58]; **glycated peptide [43-58] complexed with borate 644.988(3+)

The selective detection of glycated peptides was studied on the glycated ubiquitin hydrolysate. The whole peptic digest was incubated with phenylboronic acid in ammonium carbonate buffer, diluted with methanol, and directly analyzed on a micrOTOF instrument. The obtained results are presented in Figure 4 (the expanded range 600–825 *m/z* of obtained spectrum). The glycated peptides were identified on the basis of their characteristic isotopic pattern, comparing the spectra of the peptic mixture recorded without additives or in the presence of phenylboronate (Figure 4a, b). Our results indicate that the complexation of the phenylboronate anion by the glycated peptide may be used to distinguish glycated and non-glycated peptides. Moreover, the higher molecular mass of phenylboronic acid compared with the borate (Figure 4c) helps avoiding the overlapping of signals corresponding to the resulting complexes in complicated mixtures.

### Complex Formation Using Phenylboronic Acid QAS Derivatives

Our experiments on using borate buffer or phenylboronic acid for selective detection of sugar-peptide conjugates gave promising results. Therefore, we decided to further develop this technique. We applied our strategy to the MS analysis of trace amount of glycated peptides and non-ionizable products (or substances previously investigated only by negative ion mode MS). According to the literature, one of the known methods to increase the ionization efficiency of peptides is their derivatization to form fixed charge ionic species[39]. The combination of an efficient method of derivatization by phenylboronic acid and increased ionization efficiency by QAS should result in increase of *cis*-diols detection by positive mode ESI-MS. We present two different strategies of quaternary ammonium phenylboronic acid derivative synthesis on solid support (Figures 1 and 2). In both cases, the obtained products contain quaternary ammonium salt and phenylboronic acid moieties. To obtain PhB-K(QAS)-NH_2_ (TAG1) containing 2-(1,4-diazabicyclo[2.2.2]octylammonium)acetyl residue as QAS, the ε-amino group of lysine residue attached to the resin was first iodoacetylated. Then the product was treated with an excess of the tertiary amine. After the nucleophilic substitution of iodine atom by a tertiary amine, the Fmoc protecting group was removed from lysine and the coupling of 4-carboxyphenylboronic acid was carried out (Figure 1). The second synthetic strategy (TAG2) includes the same steps for the formation of quaternary ammonium salts, but the bifunctional 4-(dimethylamino)phenylboronic acid is used for quaternary salt formation. The iodoacetylation was performed on the free α-amino group of glycine. Then the obtained product was treated with an excess of the 4-(dimethylamino)phenylboronic acid. The extension of the peptide chain (GGG) before the QAS formation was found to improve the yield of the desired products (Figure 2). Both applied synthetic methods were based on the similar reactions, but they differ by the character of amine (tertiary aliphatic in TAG1) and substituted aniline (TAG2) as well as the order of synthetic steps. The first method requires more steps, but allows incorporation of more diverse QAS moieties.

The purity and identity of the obtained tags were confirmed by HPLC and HR-MS. The ESI-MS spectrum of the pure PhB-K(QAS)-NH_2_ is presented in Supplemental Figure S6.

Before using the synthesized tags for selective detection of *cis*-diols by mass spectrometry, preliminary experiments on ionization efficiency were conducted in water/methanol and ammonium carbonate buffer/methanol. The application of the same concentrations of tags (TAG1 and TAG2), determined according to the area under the chromatogram signal, results in a much higher intensity of the peak corresponding to cationic form of PhB-K(QAS)-NH_2_, where the quaternary ammonium salt was formed from DABCO. In separate experiments, the amounts of the compounds were adjusted to generate MS peaks of comparable intensity. It seems that the TAG2 (PhB(QAS)-GGG-NH_2_) is less ionized in ESI-MS experiments than the TAG1 (PhB-K(QAS)-NH_2_). It is possible that the differences arise from different charge distribution and hydrophobic properties of these tags. Additional experiments were performed at the same reaction conditions as to complex formation but without addition of carbohydrates or glycated compounds. The application of the same concentration of tags reveals the differences in intensity of peaks in the MS spectrum. The TAG2 shows lower intensity in mass spectrometric experiments. All investigated carbohydrates show comparable affinity to phenylboronic acid derivatives used at the same concentration (Supplemental Figure S7). Therefore, further experiments were performed both for the mixtures containing the same concentration of tags and for the mixtures producing the same intensity of their MS signals.

The lowest intensity was observed for the signals corresponding to D-glucuronic acid, probably because partial charge neutralization resulting from the carboxylic group dissociation in a basic buffer. In all cases the most abundant complex peaks correspond to the 1:1 ratio of tag–carbohydrate concentrations. When the tag concentrations were adjusted to give the same intensity of signals, after incubation with carbohydrates much higher signals were observed for TAG2 (PhB(QAS)-GGG-NH_2_) complexes, especially for D-ribose and D-2-deoxyribose (Supplemental Figure S8). It seems that the TAG2 (PhB(QAS)-GGG-NH_2_) is less ionized in mass spectrometry experiments, so to obtain the same intensity of peaks corresponding to the tags, higher concentration of PhB(QAS)-GGG-NH_2_ has to be used. The formation of the complexes with carbohydrates changes the charge distribution in TAG2 for the benefit of the fixed positive charge and the resulting product shows improved ionization efficiency. Therefore, the higher intensities of the peaks corresponding to the complexes of sugars with PhB(QAS)-GGG-NH_2_ result from the application of larger amount of this tag to get the initial comparable signal intensities. The proposed structures of the obtained complexes for D-ribose and D-2-deoxyribose with PhB(QAS)-GGG-NH_2_ and their simulated isotopic patterns are presented in Supplemental Figure S9).

A series of model glycated peptides (H-Ala-Lys(Fru)-Ala-Phe-OH, H-Asp-Thr-Glu-Lys-Gln-Ile-Lys(Fru)-Lys-Gln-Thr-OH, H-Asp-Thr-Glu-Lys(Fru)-Gln-Ile-Lys(Fru)-Lys-Gln-Thr-OH and the mixture of oligosaccharides [36] were tested for their affinity towards the phenylboronic acid derivatives.

**Figure 5:**
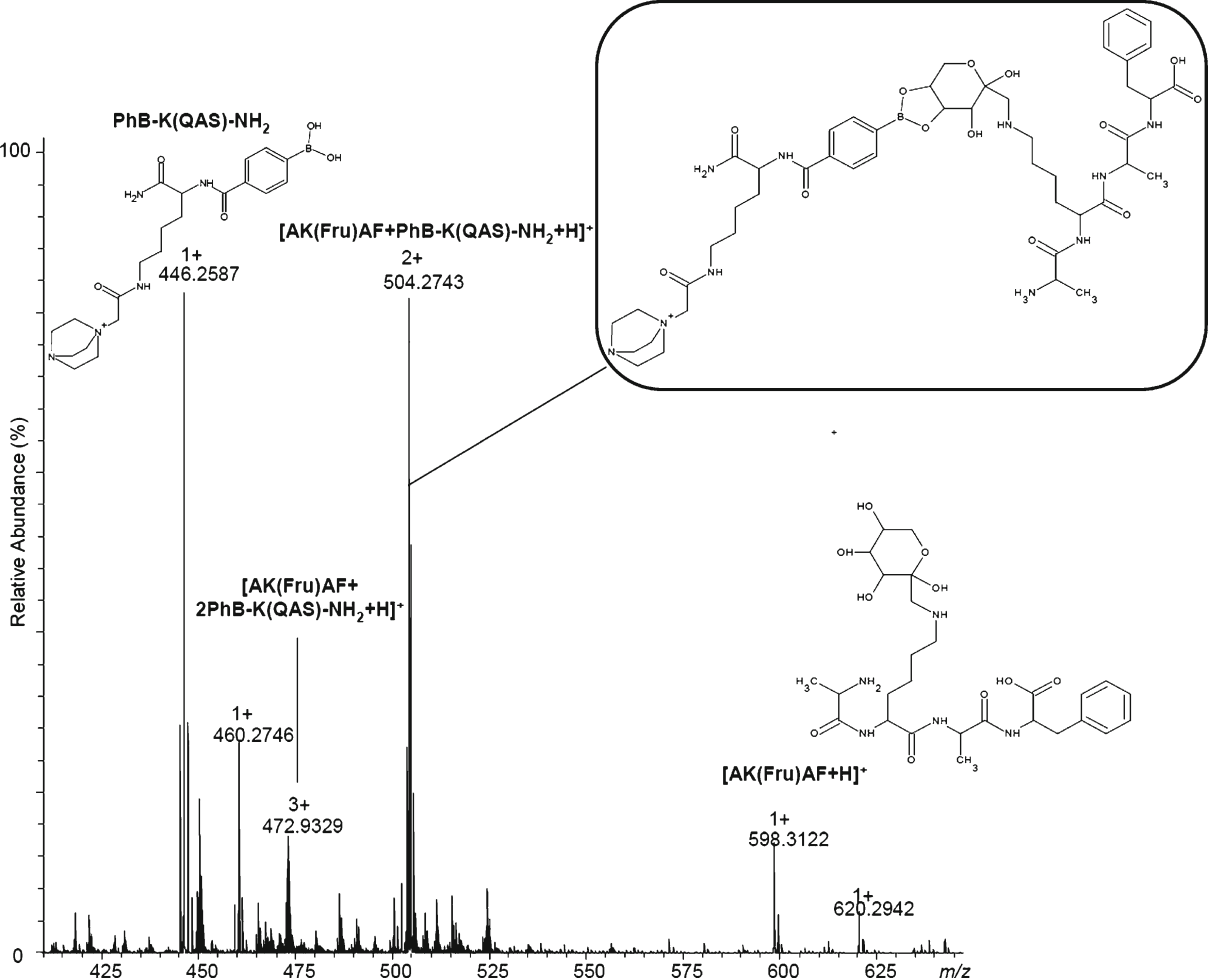
ESI-MS spectrum of the model peptide H-Ala-Lys(Fru)-Ala-Phe-OH complexed with TAG1: PhB-K(QAS)-NH_2_ (positive ion mode)

The solution of the model glycated peptide (H-Ala-Lys(Fru)-Ala-Phe-OH) was incubated with PhB-K(QAS)-NH_2_ (TAG1) in ammonium carbonate buffer, diluted with methanol and subjected to ESI-MS analysis. The most abundant complex observed by MS spectra contains one phenylboronate derivative and one molecule of the peptide; however, a complex containing two phenylboronates was also observed. Presumable structures for these complexes are placed in Supplemental Figure S10). The stoichiometry of these complexes is proven by their isotopic patterns (Figure 5). The parent ion at *m/z* 504.273 was subjected to MS/MS fragmentation using the same collision energy as in previous experiments (Supplemental Figure S11).

The fragmentation spectrum of the model glycated peptide labeled with PhB-K(QAS)-NH_2_ tag is characterized by the low abundance of ions formed by the elimination of water. The elimination of whole hexose moiety is also not observed. The fragmentation spectrum is dominated by the series of b and y ions revealing the whole sequence of peptide. The comparison of MS/MS spectra for complexes with borate, phenylboronate, and synthetic tags indicates that in this case, the largest number of b and y ions was identified.

The monoglycated and diglycated fragments of BSA (H-Asp-Thr-Glu-Lys-Gln-Ile-Lys(Fru)-Lys-Gln-Thr-OH and H-Asp-Thr-Glu-Lys(Fru)-Gln-Ile-Lys(Fru)-Lys-Gln-Thr-OH) were incubated with different amounts of PhB-K(QAS)-NH_2_ (TAG1) in ammonium carbonate buffer, diluted with methanol and subjected to ESI-MS analysis. The representative spectra are presented in Supplemental Figures S12 and S13. The ESI spectra indicate the interactions of the peptide with phenylboronate derivative. A series of complexes containing one phenyboronate anion are observed [597.3042 (3+) for monoglycated peptide; 651.343 (3+) for diglycated peptide], however, a complex containing two phenylboronate ions was also observed for peptide containing two hexose moieties (Supplemental Figure S13). The application of increased concentrations of the tag does not lead to complete labeling of glycated peptide contrary to what was observed for model glycated tetrapeptide. The repulsion of positive charges located on the peptide molecule may be responsible for this phenomenon. The intensity of the observed complex ion is high enough to easily identify it in the MS spectrum.

The mixture of mono- and diglycated peptides was incubated with the mixture of tags. The tags were used in the amounts selected to obtain the initial MS peaks of comparable intensity. Supplemental Figures S14 shows a series of mixed complexes containing one or two phenylboronate derivatives. The affinity of tags for monoglycated and diglycated peptides is comparable because the intensity of peaks corresponding to the complexes is similar.

**Figure 6:**
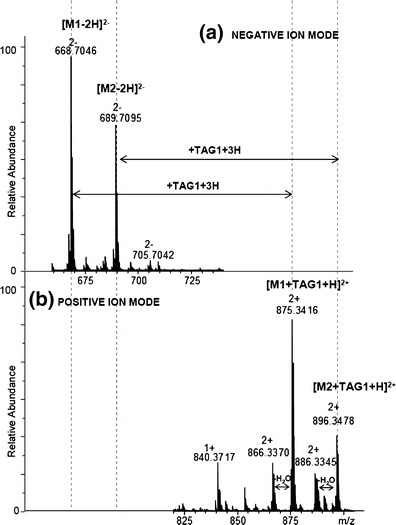
ESI-MS spectra of the oligosaccharides isolated from *H. alvei* LPS built of one RU linked to the Hep-Kdo disaccharide: (**a**) sample dissolved in ammonium carbonate buffer (negative ion mode); (**b**) sample with TAG1 dissolved in ammonium carbonate (positive ion mode); (M1, M2: 3-deoxy-D*-manno*-oct-2-ulosonic acid – Kdo; repeating unit – RU; L-glycero-D-*manno*-heptose – Hep, Ac – O-acetyl group)

The formation of the phenylboronate derivatives complexes was also studied on the oligosaccharide isolated from *Hafnia alvei* PCM (Polish Collection of Microorganisms) 1200 lipopolysaccharide (LPS). *H. alvei* is a gram-negative bacterium and a causative agent of respiratory diseases, mixed hospital infections, bacteremia, and septicemia in humans [36]. The oligosaccharide is composed of one repeating unit (RU) of the LPS 1200 O-specific polysaccharide devoid of terminal Glc residue (Qui*p*4NAc + GroP + Gal + 2xGlc*p*NAc) linked to the Hep-Kdo disaccharide. It is the shortest form of the O-specific chain present on bacterial surface. Two variants were identified—substituted with and devoid of one O-acetyl group (Ac). Before incubation with phenyboronate derivative (TAG1 - PhB-K(QAS)-NH_2_), the oligosaccharide may be analyzed only in negative ion mode MS (Figure 6a). After the incubation, the oligosaccharides may also be investigated in positive ion mode MS (Figure 6b). In the spectrum, the series of complexes are observed (Figure 6b). The isotopic patterns of these complexes are typical for compounds containing boron atom.

## Conclusions

The new tags based on the derivatives of phenylboronic acid were designed, synthesized according to the solid phase Fmoc-strategy, and applied for selective detection of sugars and peptide-sugar conjugates in mass spectrometry. The formation of complexes between sugar or sugar-peptide conjugates with reagents (borate, phenylboronate, phenylboronate derivatives) was confirmed on the basis of the unique isotopic distribution resulting from the presence of boron atom. The straightforward and convenient method based on the formation of borate and phenylboronate complexes of carbohydrates and peptide-derived Amadori products and their specific behavior in CID experiments was developed. For all reagents (borate, phenylboronate and phenylboronate derivatives), the stabilization of hexose moiety in MS/MS was observed. The neutral losses are significantly reduced in respect to free peptide-derived Amadori products; therefore the fragmentation spectra are simplified. The complexation of the phenylboronate or borate ion by the glycated peptide may be used to distinguish glycated and non-glycated peptides. Moreover, the higher molecular mass of phenylboronic acid compared with the borate limits the risk of overlapping signals corresponding to the resulting complexes in complicated mixtures. Unfortunately, interactions of glycated peptides with QAS-containing phenylboronate tags did not result in significant increase in sensitivity of glycated peptides detection. On the other hand, the ionization of monosacharides as well as natural mixture of lipoplysacharides in positive ion mode was enhanced. The combination of strategies involving phenylboronic acids and QAS is a promising method for sensitive detection of carbohydrates by electrospray ionization tandem mass spectrometry. Moreover, studies on sugars–phenylboronic acid interactions may be useful for the development of carbohydrate receptors.

## Electronic supplementary material

Below is the link to the electronic supplementary material.

ESM 1 (DOC 884 kb)
