# Supplementary material for: Selective Detection of Carbohydrates and Their Peptide Conjugates by ESI-MS Using Synthetic Quaternary Ammonium Salt Derivatives of Phenylboronic Acids
Source: J Am Soc Mass Spectrom. 2014 Apr 1;25(6):966–76. doi: 10.1007/s13361-014-0857-4 (PMC4018510; doi:10.1007/s13361-014-0857-4)
Supplement: Supplementary file 1 — (DOC 884 kb) [file 13361_2014_857_MOESM1_ESM.doc]

Supplementary data

Journal of the American Society for Mass Spectrometry

Selective detection of carbohydrates and their peptide conjugates by ESI-MS using QAS synthetic derivatives of phenylboronic acids

Monika Kijewska^1^, Adam Kuć^1^, Alicja Kluczyk^1^, Mateusz Waliczek^1^, Aleksandra

Man-Kupisinska^2^, Jolanta Lukasiewicz^2^, Piotr Stefanowicz^1^, Zbigniew Szewczuk^1^

*^1)^ Faculty of Chemistry, University of Wrocław, Wrocław, Poland*

*^2)^ Department of Immunochemistry, Ludwik Hirszfeld Institute of Immunology and Experimental Therapy, Polish Academy of Sciences, Wroclaw, Poland*

Running title: Phenylboronic QAS tags for carbohydrates

Corresponding author: Monika Kijewska, Faculty of Chemistry, University of Wrocław, F. Joliot-Curie 14, 50-383 Wrocław, Poland, Fax: +48 71 3282348, Tel.: +48-71-3757213, E mail: monika.kijewska@chem.uni.wroc.pl

Fig. S1 MS/MS spectrum of the Fmoc-Lys(Fru)-OH complexed with phenylboronic acid (positive ion mode; parent ion of free Fmoc-Lys(Fru)-OH 531.234 *m/z*; collision energy 5-25eV)‏

Fig. S2 MS/MS spectrum of the Fmoc-Lys(Fru)-OH complexed with phenylboronic acid (positive ion mode; parent ion 617.269 *m/z*; collision energy 5-35eV)‏

Fig. S3 MS/MS spectrum of the Fmoc-Lys(Fru)-OH complexed with phenylboronic acid (positive ion mode; parent ion 703.304 *m/z*; collision energy 10-35eV)‏

Fig. S4 ESI-MS spectrum of the model peptide H-Ala-Lys(Fru)-Ala-Phe-OH complexed with phenylboronic acid (PhB) (positive ion mode)‏. (Bottom panel - simulated isotopic patterns for molecular formula of investigated compounds).

**a)**

**b)**

Fig. S5 a) MS/MS spectrum of the glycated peptide AK(Fru)AF complexed with phenylboronic acid ((positive ion mode; parent ion 684.35 *m/z*; collision energy 10-45eV)‏ b) MS/MS spectrum of the glycated peptide AK(Fru)AF complexed with phenylboronic acid (positive ion mode; parent ion 684.35 *m/z*; collision energy 20eV)‏; asterisks represents elimination of a whole hexose moiety.

Fig. S6 ESI-MS spectrum of pure TAG1 (PhB-K(QAS)-NH_2_)

Fig. S7 ESI-MS spectrum of the mixture of carbohydrates (D-ribose, D-glucuronic acid, D-2-deoxyribose, N-acetylglucosamine, saccharose) complexed with equimolar mixture of tags: TAG1 (PhB-K(QAS)-NH_2_) (purple) and TAG2 (PhB(QAS)-GGG-NH_2_) (green)

Fig. S8 ESI-MS spectrum of the mixture of carbohydrates (D-ribose, D-glucuronic acid, D-2-deoxyribose, N-acetylglucosamine, saccharose) complexed with synthetic tags: TAG1 (PhB-K(QAS)-NH_2_) (purple) and TAG2 (PhB(QAS)-GGG-NH_2_) (green) (the same intensity of initial tag peaks)

Fig. S9 ESI-MS spectrum of D-ribose and D-2-deoxyribose complexed with TAG2 (PhB(QAS)-GGG-NH_2_) (expanded rage of spectrum; bottom panel - simulated isotopic patterns for the proposed molecular formula of the investigated compounds)

Fig. S10 Presumable structures of AK(Fru)AF complexed with TAG1 (PhB-K(QAS)-NH_2_)

Fig. S11 MS/MS spectrum of the glycated peptide AK(Fru)AF complexed with TAG1 (PhB-K(QAS)-NH_2_) (positive ion mode; parent ion 504.27 m/z; collision energy 20eV)‏

Fig. S12 ESI-MS spectrum of monoglycated peptide (H-Asp-Thr-Glu-Lys-Gln-Ile-Lys(Fru)-Lys-Gln-Thr-OH) complexed with TAG1 (PhB-K(QAS)-NH_2_) (positive ion mode)

Fig. S13 ESI-MS spectrum of diglycated peptide (H-Asp-Thr-Glu-Lys(Fru)-Gln-Ile-Lys(Fru)-Lys-Gln-Thr-OH) complexed with TAG1 (PhB-K(QAS)-NH_2_) (positive ion mode)

Fig. S14 ESI-MS spectrum of mixture of mono (M1) and diglycated (M2) peptides complexed with TAG1 (PhB-K(QAS)-NH_2_) and TAG2 (PhB(QAS)-GGG-NH_2_) (positive ion mode)

**Figure captions**

[**Fig. S1** MS/MS spectrum of the Fmoc-Lys(Fru)-OH complexed with phenylboronic acid (positive ion mode; parent ion of free Fmoc-Lys(Fru)-OH 531.234 *m/z*; collision energy 5-25eV)‏](#__RefHeading___Toc379280325)

[**Fig. S2** MS/MS spectrum of the Fmoc-Lys(Fru)-OH complexed with phenylboronic acid (positive ion mode; parent ion 617.269 *m/z*; collision energy 5-35eV)‏](#__RefHeading___Toc379280326)

[**Fig. S3** MS/MS spectrum of the Fmoc-Lys(Fru)-OH complexed with phenylboronic acid (positive ion mode; parent ion 703.304 *m/z*; collision energy 10-35eV)‏](#__RefHeading___Toc379280327)

[**Fig. S4** ESI-MS spectrum of the model peptide H-Ala-Lys(Fru)-Ala-Phe-OH complexed with phenylboronic acid (PhB) (positive ion mode)‏. (Bottom panel - simulated isotopic patterns for molecular formula of investigated compounds).](#__RefHeading___Toc379280328)

[**Fig. S5** a) MS/MS spectrum of the glycated peptide AK(Fru)AF complexed with phenylboronic acid ((positive ion mode; parent ion 684.35 *m/z*; collision energy 10-45eV)‏ b) MS/MS spectrum of the glycated peptide AK(Fru)AF complexed with phenylboronic acid (positive ion mode; parent ion 684.35 *m/z*; collision energy 20eV)‏; asterisks represents elimination of a whole hexose moiety.](#__RefHeading___Toc379280329)

[**Fig. S6** ESI-MS spectrum of pure TAG1 (PhB-K(QAS)-NH_2_)](#__RefHeading___Toc379280330)

[**Fig. S7** ESI-MS spectrum of the mixture of carbohydrates (D-ribose, D-glucuronic acid, D-2-deoxyribose, N-acetylglucosamine, saccharose) complexed with equimolar mixture of tags: TAG1 (PhB-K(QAS)-NH_2_) (purple) and TAG2 (PhB(QAS)-GGG-NH_2_) (green)](#__RefHeading___Toc379280331)

[**Fig. S8** ESI-MS spectrum of the mixture of carbohydrates (D-ribose, D-glucuronic acid, D-2-deoxyribose, N-acetylglucosamine, saccharose) complexed with synthetic tags: TAG1 (PhB-K(QAS)-NH_2_) (purple) and TAG2 (PhB(QAS)-GGG-NH_2_) (green) (the same intensity of initial tag peaks)](#__RefHeading___Toc379280332)

[**Fig. S9** ESI-MS spectrum of D-ribose and D-2-deoxyribose complexed with TAG2 (PhB(QAS)-GGG-NH_2_) (expanded rage of spectrum; bottom panel - simulated isotopic patterns for the proposed molecular formula of the investigated compounds)](#__RefHeading___Toc379280333)

[**Fig. S10** Presumable structures of AK(Fru)AF complexed with TAG1 (PhB-K(QAS)-NH_2_)](#__RefHeading___Toc379280334)

[**Fig. S11** MS/MS spectrum of the glycated peptide AK(Fru)AF complexed with TAG1 (PhB-K(QAS)-NH_2_) (positive ion mode; parent ion 504.27 m/z; collision energy 20eV)‏](#__RefHeading___Toc379280335)

[**Fig. S12** ESI-MS spectrum of monoglycated peptide (H-Asp-Thr-Glu-Lys-Gln-Ile-Lys(Fru)-Lys-Gln-Thr-OH) complexed with TAG1 (PhB-K(QAS)-NH_2_) (positive ion mode)](#__RefHeading___Toc379280336)

[**Fig. S13** ESI-MS spectrum of diglycated peptide (H-Asp-Thr-Glu-Lys(Fru)-Gln-Ile-Lys(Fru)-Lys-Gln-Thr-OH) complexed with TAG1 (PhB-K(QAS)-NH_2_) (positive ion mode)](#__RefHeading___Toc379280337)

[**Fig. S14** ESI-MS spectrum of mixture of mono (M1) and diglycated (M2) peptides complexed with TAG1 (PhB-K(QAS)-NH_2_) and TAG2 (PhB(QAS)-GGG-NH_2_) (positive ion mode)](#__RefHeading___Toc379280338)

**Alternative version of Figure 6 is included in the manuscript**

Fig. 1 ESI-MS spectra of the oligosaccharides isolated from H. alvei LPS built of one RU linked to the Hep-Kdo disaccharide: a) sample dissolved in ammonium carbonate buffer (negative ion mode); b) sample with TAG1 dissolved in ammonium carbonate (positive ion mode);

(M1, M2: 3-deoxy-D-manno-oct-2-ulosonic acid - Kdo; repeating unit – RU; L-glycero-D-manno-heptose – Hep, Ac – O-acetyl group)
